# Generation and Characterization of Novel Magnetic Field-Responsive Biomaterials

**DOI:** 10.1371/journal.pone.0133878

**Published:** 2015-07-24

**Authors:** Modesto T. Lopez-Lopez, Giuseppe Scionti, Ana C. Oliveira, Juan D. G. Duran, Antonio Campos, Miguel Alaminos, Ismael A. Rodriguez

**Affiliations:** 1 Department of Applied Physics, Faculty of Sciences, University of Granada, Granada, Spain, and Instituto de Investigación Biosanitaria ibs.GRANADA, Granada, Spain; 2 Department of Histology, Faculty of Medicine, University of Granada, Granada, Spain, and Instituto de Investigación Biosanitaria ibs.GRANADA, Granada, Spain; 3 Department of Histology, School of Dentistry, National University of Cordoba, Cordoba, Argentina; National University of Ireland, Galway (NUI Galway), IRELAND

## Abstract

We report the preparation of novel magnetic field-responsive tissue substitutes based on biocompatible multi-domain magnetic particles dispersed in a fibrin–agarose biopolymer scaffold. We characterized our biomaterials with several experimental techniques. First we analyzed their microstructure and found that it was strongly affected by the presence of magnetic particles, especially when a magnetic field was applied at the start of polymer gelation. In these samples we observed parallel stripes consisting of closely packed fibers, separated by more isotropic net-like spaces. We then studied the viability of oral mucosa fibroblasts in the magnetic scaffolds and found no significant differences compared to positive control samples. Finally, we analyzed the magnetic and mechanical properties of the tissue substitutes. Differences in microstructural patterns of the tissue substitutes correlated with their macroscopic mechanical properties. We also found that the mechanical properties of our magnetic tissue substitutes could be reversibly tuned by noncontact magnetic forces. This unique advantage with respect to other biomaterials could be used to match the mechanical properties of the tissue substitutes to those of potential target tissues in tissue engineering applications.

## Introduction

Biomaterials intended for applications in regenerative medicine must imitate the histological structure of natural tissues. They should thus meet a number of requirements, including biocompatibility [1–4]. Various scaffold materials have been tested, including both naturally-derived and synthetic polymers. Although natural materials provide a physiological environment for cell adhesion and proliferation, they have several disadvantages, such as their suboptimal mechanical properties [5–8]. Synthetic materials are extensively used because of their easy molding characteristics, relatively easy production and their ability to control dissolution and degradation [9]. The main drawback of synthetic materials is that they do not have natural sites for cell adhesion [10].

One alternative to choosing between natural or synthetic materials is to use them in combination [11]. For example, several authors have very recently used magnetic nanoparticles in combination with polymers to prepare innovative magnetic scaffolds for tissue substitutes [12–29]. These magnetic scaffolds have several advantages. First, the ferromagnetic behavior of the magnetic scaffolds allows visualization and in-vivo follow-up by magnetic resonance imaging [28]. Second, in-vitro studies indicate that magnetic nanoparticles in the scaffolds do not compromise cell adhesion, proliferation or differentiation [12,13,25]. Furthermore, the main advantage of novel magnetic scaffolds is that they acquire a magnetic moment when an external magnetic field is applied, i.e. they act as magnets, attracting functionalized magnetic nanoparticles injected close to them [13,21,28]. This represents a promising strategy to guide and accumulate growth factors, drugs and cells previously attached to the injected magnetic nanoparticles.

To the best of our knowledge, all magnetic scaffolds described to date are based on the use of magnetic particles measuring on the order of 10 nm in diameter. Magnetic particles of this size are single-domain in terms of their magnetic behavior. Moreover, because of their small size the magnetic energy of interaction between particles is weak compared to the energy of Brownian motion [30]. As a result, even for strong applied magnetic fields, Brownian motion dominates over the magnetic forces, and the mechanical properties of the scaffolds cannot be controlled by noncontact magnetic forces. The situation is radically different for magnetic particles larger than approximately 50–100 nm. Particles of this size are multi-domain in terms of their magnetic behavior. This means that there is no magnetic interaction between them prior to the application of a magnetic field. In addition, because of their relatively large size, Brownian motion is negligible compared to magnetic interaction in the presence of moderate magnetic fields [30], which makes it theoretically possible to control, via noncontact magnetic forces, the mechanical properties of biomaterials that contain the particles.

The main aim of the present study was to generate magnetic biomaterials whose mechanical properties can be controlled by noncontact magnetic forces. To this end we used a mixture of fibrin and agarose as a polymer matrix. We chose this combination because fibrin is a natural polymer used frequently in tissue engineering. The main drawback of fibrin hydrogels lies in their suboptimal biomechanical properties, which fortunately can be enhanced by combining them with agarose [31]. We previously showed that these fibrin–agarose biomaterials have better biomechanical and structural properties than fibrin alone [31–33]. In addition, we recently demonstrated that the biomechanical properties of fibrin–agarose hydrogels reproduce the properties of several native soft human tissues [31]. Fibrin–agarose biomaterials have been used successfully to generate bioengineered substitutes of several human tissues such as the cornea, oral mucosa, skin and peripheral nerves, and were shown to be effective in vivo [32–34]. In the present study we demonstrate that the incorporation of magnetic particles gives rise to bioengineered oral mucosa tissue substitutes with a tunable, reversible mechanical response. In tissue engineering applications this versatility should make it possible to adjust the mechanical properties of the artificial tissue substitutes with precision, in order to match the properties of the target tissue at the site of implantation.

## Materials and Methods

### Ethics statement

This study was approved by the Ethics Committee of the University of Granada, Granada, Spain. Each tissue donor signed an informed consent form for this study.

### Establishment of primary cultures of oral mucosa fibroblasts

Ten normal human oral mucosa biopsies with an average volume of 8 mm^3^ were obtained from healthy donors at the School of Dental Sciences of the University of Granada. To obtain primary cultures of human oral mucosa fibroblasts, tissues were enzymatically de-epithelized and the lamina propria was digested in a mixture of Dulbecco’s Modified Eagle’s Medium (DMEM) and 2 mg/mL *Clostridium histolyticum* collagenase I (Gibco BRL Life Technologies, Karlsruhe, Germany). Detached fibroblasts were collected by centrifugation and expanded in culture fiasks containing DMEM supplemented with 10% fetal calf serum (FCS) and 1% antibiotic–antimycotic solution (final concentration 100 U/mL penicillin G, 0.10 mg/mL streptomycin and 0.25 μg/mL amphotericin B) (all from Sigma-Aldrich, Steinheim, Germany). Cells were incubated at 37°C in 5% carbon dioxide under standard culture conditions. The medium was changed every 3 days, and the cells were subcultured in a solution of 0.5 g/L trypsin and 0.2 g/L EDTA at 37°C for 10 min. For all experiments we used cells from the first 3 passages of these human oral mucosa fibroblast cell cultures.

### Preparation of the biomaterials (three-dimensional tissue substitutes)

For the magnetic phase we used MagP-OH particles (Nanomyp, Granada, Spain). According to the manufacturer, MagP-OH particles consist of biocompatible nanoparticles with a mean diameter of 115 nm, comprising a single magnetic core of magnetite (γ-Fe_3_O_4_) coated by a polymer layer of methyl methacrylate-co-hydroxyl ethyl methacrylate-co-ethylene glycol dimethacrylate. MagP-OH particles were supplied as an aqueous suspension stabilized with surfactants, and were treated before use with 5 washing cycles (centrifugation at 15000 g for 30 min, supernatant discarded, ultrapure water added, particles redispersed) to remove the surfactant. We then replaced the water carrier with 70% ethanol and left the nanoparticles in this solution for 12 h for sterilization. Finally the ethanol was removed, and the nanoparticles were suspended in DMEM.

For the continuous matrix we used a mixture of fibrin and agarose as the biopolymer. The target tissue was human oral mucosa, thus, seeding with human oral mucosa fibroblasts was required. To prepare the magnetic tissue substitutes we used a variation of a previous method for fibrin–agarose nonmagnetic scaffolds [32]. Briefly, we used 3.8 mL human plasma obtained from blood donors (provided by the Granada Biobank of the Andalusian Regional Government), to which we added 1,000,000 oral mucosa fibroblasts resuspended in 0.625 mL DMEM, together with 75 μL of a solution of tranexamic acid at a concentration of 0.1 g/mL. The final concentration of tranexamic acid in the biomaterial was 1.5 mg/mL. This acid is an anti-fibrinolytic agent that prevents degradation of the scaffold. We then added the appropriate amounts of a concentrated suspension of MagP-OH particles in DMEM to a final concentration of approximately 2 mL of particles per 100 mL of mixture. Subsequently, 0.25 mL of a mixture of type VII agarose (a polysaccharide polymer material with a molecular weight of approx. 120,000 g/mol, supplied by Sigma-Aldrich Química SA, Madrid, Spain) in phosphate-buffered saline (PBS) (0.02 g/mL concentration) was added to the mixture to a final agarose concentration of 0.1%. Then 0.25 mL of 2% CaCl_2_ was added to the mixture to activate the fibrin polymerization process. The final volume of the mixture was 5 mL, which contained 200,000 cells per mL of mixture. This cell density is on the same order of magnitude as (or higher than) the number of cells validated for fibrin–agarose gels in previous studies [32–34].

The mixtures were seeded on petri dishes and kept at 37°C for 2 h until gelation was complete. We applied a vertical magnetic field to the mixtures during the first 5 min of gelation with a coil connected to a DC power supply. We subjected different samples to different field strengths ranging from 0 to 48 kA/m in intensity (0, 16, 32 or 48 kA/m). After 2 h we added to the gels (tissue substitutes) DMEM medium supplemented with 10 vol% FCS and 1 vol% antibiotic–antimycotic solution, and incubated the cultures for 24 h at 37°C in the culture dishes.

For comparison we also prepared nonmagnetic tissue substitutes (control samples) with the same procedure as described above, except for the addition of magnetic particles. We also subjected these samples to applied magnetic fields of different intensity during gelation (0, 16, 32 or 48 kA/m) in order to analyze the effect of the magnetic field on the biological constituents in the tissue substitutes. To analyze the effect of the magnetic MagP-OH particles on the substitute properties more precisely, we also prepared a nanoparticle control sample (Ctrl-NP) which contained nonmagnetic polymer particles. These particles (PolymP-C, NanoMyP) were uniformly spherical and similar in diameter (approximately 130 nm) to MagP-OH particles, but lacked magnetic properties. Their chemical composition was similar to the polymer layer constituting the shell of MagP-OH, since they are made from the same polymers with OH functionalization. We prepared Ctrl-NP tissue substitutes with the same procedure as described above for magnetic tissue substitutes, but with PolymP-C particles instead of MagP-OH particles. Prior to use we sterilized the PolymP-C particles by immersion for 12 h in 70% ethanol, followed by ethanol removal and dispersion in DMEM.

In all, we prepared oral mucosa substitutes with 9 different protocols (Table 1). The density of all substitutes was approximately 1.1 g/mL.

**Table 1:** Summary of the different oral mucosa substitutes prepared for this study.

Magnetic field strength during gelation (kA/m)	Approx. concentration of particles (volume %)	Type of particles	Sample name
0	0	—	Ctrl-MF0
0	2	MagP-OH ª	M-MF0
0	2	PolymP-C ^b^	Ctrl-NP
16	0	—	Ctrl-MF16
16	2	MagP-OH ª	M-MF16
32	0	—	Ctrl-MF32
32	2	MagP-OH ª	M-MF32
48	0	—	Ctrl-MF48
48	2	MagP-OH ª	M-MF48

ª Magnetite (core)/polymer (shell) MagP-OH particles (Nanomyp).

^b^ Nonmagnetic polymer PolymP-C particles (Nanomyp).

### Structural analysis

After 24 h of cell culture, we fixed the oral mucosa tissue substitutes in formalin, embedded them in paraffin, and cut them in 5-μm-thick sections. After deparaffination and hematoxylin–eosin staining, we analyzed structural features in the biomaterial sections by light microscopy. For scanning electron microscopy (SEM), samples were fixed in 2.5% glutaraldehyde and postfixed in 1% osmium tetroxide for 90 min. After fixation, the samples were dehydrated in increasing concentrations of acetone (30%, 50%, 70%, 95% and 100%), critical point-dried, mounted on aluminum stubs and sputter-coated with gold according to routine procedures.

### Cell viability analysis

We evaluated cell viability by measuring intracellular esterase activity, and by examining the integrity of the plasma and nuclear membranes with a fluorescence-based method using the Live/Dead commercial kit (Life Technologies, Carlsbad, CA, USA). This method uses calcein-AM, which is metabolically modified by living cells to a green pigment, and ethidium homodimer-1, which stains the nuclei of dead cells red. We obtained aliquots of 3 mm in diameter of all biomaterial samples after 24 h of cell culture, discarded the supernatants and washed the aliquots with PBS, then cut them into very thin films, incubated them with the Live/Dead solution for 15 min as indicated by the manufacturer, and washed them with PBS. We then observed the samples by fluorescence microscopy and processed the images with ImageJ software to quantify the number of live (green) and dead cells (red).

We also evaluated cell death as nuclear membrane integrity by quantifying the DNA released to the culture medium. We obtained supernatants of each sample and diluted 10-μL aliquots in distilled water free of nuclease (Ambion-Life Technologies, Austin, TX, USA). The DNA in the medium was quantified spectrophotometrically (SmartSpec Plus, Bio-Rad, Hercules, CA, USA) at wavelengths in the range of 260–280 nm.

The mean values ± standard deviations of 8 independent experiments are reported here for each experimental group and each analysis. The Kruskal–Wallis test was used to identify statistical differences among study groups, and the Mann–Whitney test was used to identify significant differences between two groups. Values of p less than 0.05 were considered statistically significant in two-tailed tests.

### Magnetic properties

We measured the magnetization (*M*) of dry MagP-OH particles at 37°C as a function of the magnetic field strength (*H*) in a Squid Quantum Design MPMS XL magnetometer. In addition, we obtained the magnetization curve of soaked tissue substitutes 24 h after cell culture. The magnetization curves reported here correspond to the mean of 3 independent measurements.

### Mechanical properties

We characterized the mechanical properties of the tissue substitutes (summarized in Table 1) with a Haake MARS III (Thermo Fisher Scientific, Waltham, MA, USA) controlled stress rheometer at 37°C. The measuring system geometry was a 3.5 cm diameter parallel plate set with rough surfaces to avoid wall slip.

We obtained measurements as follows. First we placed the sample in the rheometer measuring system and squeezed it by lowering the rotating plate until a normal force of 5 N was reached. The rheometer gap at which this force was seen varied slightly depending on the sample, but was in all cases approximately 300 μm. We obtained measurements both in the absence and presence of a magnetic field. For this purpose we used a coil connected to a DC power supply, with the axis of the coil aligned with the axis of the parallel plate measuring system. For measurements obtained during magnetic field application, we applied the magnetic field from 1 min before measurement was started until the measurement was recorded. We used two types of rheological test: oscillatory shear at a fixed frequency, and steady-state shear strain ramps, as described below.

#### Oscillatory shear in fixed frequency–variable amplitude sweeps

For these tests, we subjected the samples to sinusoidal shear strains at a fixed frequency (1 Hz) and increasing amplitude (logarithmically spaced in the 0.05–1.0 range), and measured the corresponding oscillatory shear stresses. Each frequency–amplitude pair was maintained during 5 oscillatory cycles, although we only used data for the last 3 cycles to rule out transients. These measurements were used to calculate the elastic modulus G′ as a function of shear strain.

#### Steady state shear strain ramps

In these tests the samples were subjected to a constant shear strain for 10 s and the resulting shear stress was measured. Measurements were repeated at increasing (linearly spaced) shear strain values until the nonlinear regime was reached.

We carried out each type of measurement for 3 different aliquots of each sample. For each aliquot we carried out at least 3 repetitions to record a minimum of 9 values per data point. First we recorded measurements at *H* = 0 kA/m, then at *H* = 9 kA/m, *H* = 17 kA/m, and *H* = 26 kA/m. Then we returned to *H* = 0 kA/m and repeated this cycle at least twice for each aliquot. The results obtained for each sample and experimental condition showed no statistically significant differences.

## Results and Discussion

### Structural analysis

Macroscopically, the magnetic tissue substitutes (M-MF0, M-MF16, M-MF32, M-MF48) were similar in appearance to nonmagnetic tissue substitutes (Ctrl-MF0, Ctrl-MF16, Ctrl-MF32, Ctrl-MF48, Ctrl-NP), although the former were darker than control tissue substitutes without particles (Ctrl-MF0 to Ctrl-MF48), which were whitish and semitransparent, and control tissue substitutes with nonmagnetic particles (Ctrl-NP), which were bright white. Magnetic tissue substitutes were attracted by a magnet, as seen in S1 Video.

**Fig 1:**
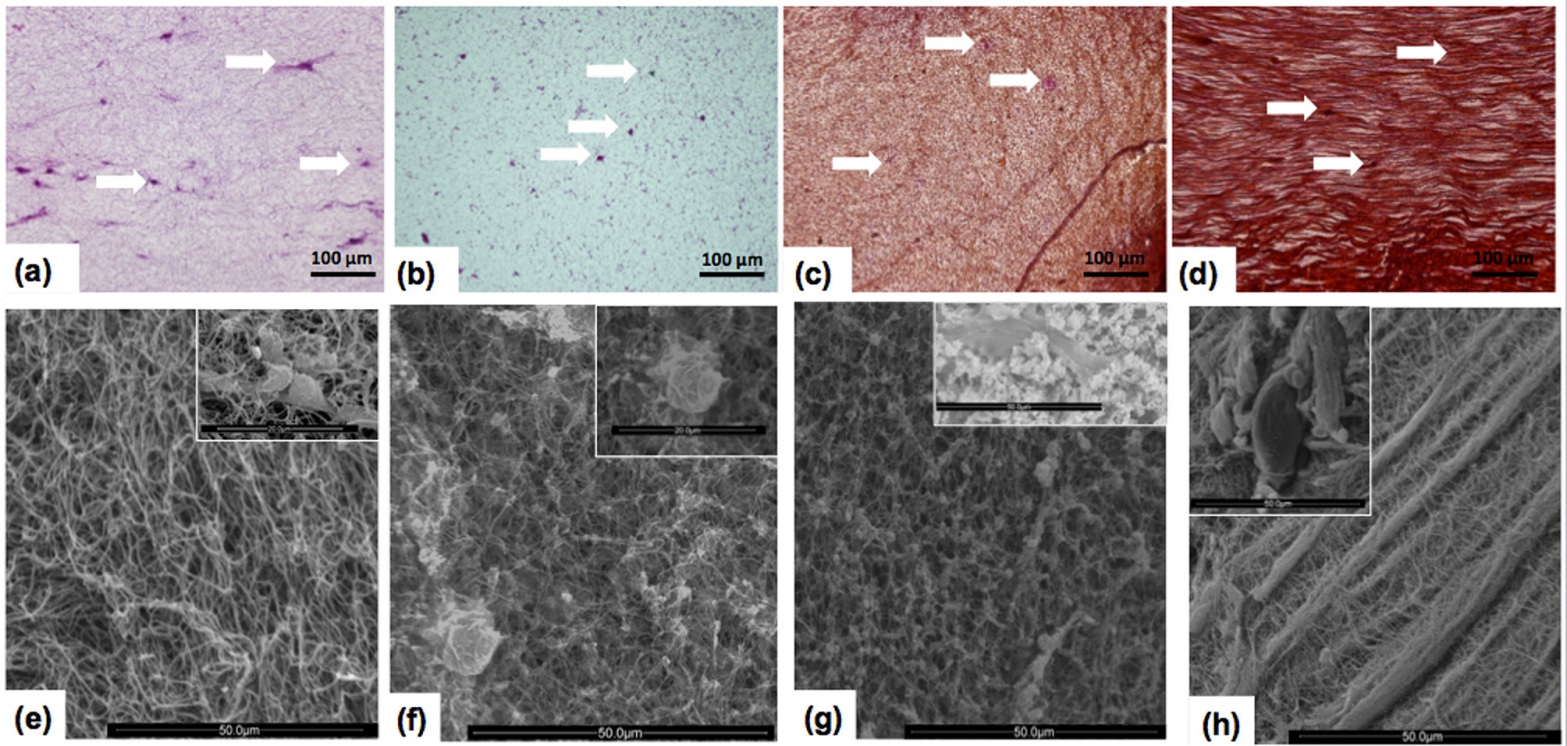
Microscopic images of tissue substitutes. 1a-1d: Light microscopy. 1e-1h: Scanning electron micrographs with individual cells shown in the insets. 1a and 1e: control (nonmagnetic) samples gelled in the absence of a magnetic field (Ctrl-MF0). 1b and 1f: control (nonmagnetic) samples with nonmagnetic polymer particles (Ctrl-NP). 1c and 1g: magnetic samples gelled in the absence of a magnetic field (M-MF0). 1d and 1h: magnetic samples gelled during application of a magnetic field (32 kA/m in 1d, and 48 kA/m in 1h). A few of the cells are marked with arrows in Fig 1a to 1d. Scale bars: Fig 1a-1d, 100 μm; Fig 1e-1h, 50 μm including insets, except for insets in Fig 1e and 1f, 20 μm.

For the control group without particles gelled in the absence of an applied magnetic field (Ctrl-MF0), microscopic analysis showed normally-shaped fusiform and star-shaped cells (Fig 1A). The cells were distributed throughout the fibrin–agarose matrix in a normal, net-like appearance. There were no cell–cell contacts, but cell–matrix contacts were evident, as expected in a connective tissue substitute. Cells in the control groups without particles gelled in the presence of an applied magnetic field were similar in appearance (not shown). In samples containing particles, we found that in the magnetic tissue substitute gelled in the absence of an applied magnetic field (M-MF0), as well as the control tissue substitute with nonmagnetic polymer particles (Ctrl-NP), the particles were distributed randomly in an isotropic, homogeneous pattern (Fig 1B and 1C). In contrast, magnetic samples gelled in the presence of a magnetic field (M-MF16, M-MF32, and M-MF48) presented a microscopic pattern consisting of filament-like structures aligned in the same direction, regardless of the intensity of the applied field (Fig 1D).

Scanning electron micrographs showed that control samples without particles (Ctrl-MF0 to Ctrl-MF48) presented an isotropic, homogeneous network of randomly aligned fibrin fibers (see Ctrl-MF0, Fig 1E). Application of a magnetic field during gelation in these control samples did not lead to significant changes in their microscopic morphology. Samples Ctrl-MF16 to Ctrl-MF48 (not shown) were similar in appearance to Ctrl-MF0. The presence of magnetic or nonmagnetic nanoparticles induced changes in the fibrillar pattern even in the absence of a magnetic field during gelation. Although the tissue substitutes retained their homogeneous morphology, some particles and particle aggregates were homogeneously distributed throughout the fibrin network, disrupting its mesoscopic ordering (Fig 1F and 1G). When a magnetic field was applied during gelation in magnetic samples, the fibrin network presented an anisotropic pattern (with one direction predominating) characterized by thick stripes containing closely packed fibrin fibers aligned and braided in the direction of the stripes, and isotropic net-like spaces between the stripes, with fewer fibers (Fig 1H, M-MF48). The stronger the field applied during gelation, the more evident the thick stripes. At the highest field strength (sample M-MF48) these stripes were 3.2 ± 1.3 μm in diameter. The aligned distribution of fibers associated with the formation of stripes might induce contact guidance of cells.

The reasons for the striped appearance of magnetic tissue substitutes gelled during exposure to a magnetic field merit consideration. To prepare samples M-MF16, M-MF32 and M-MF48 we applied a magnetic field from the beginning of gelation for 5 min. Application of a magnetic field to multi-domain magnetic particles (such as MagP-OH nanoparticles) induces the appearance of a net magnetic moment aligned with the field direction in each particle (i.e., polarization of the particle). This results in magnetostatic forces of attraction between particles, and when particles are free to move (i.e., when they are dispersed in a liquid-like carrier), they migrate and aggregate into chain-like structures aligned with the field direction, in order to minimize the energy of the system [30]. Since the speed of particle polarization and migration is on the order of milliseconds [30], it is reasonable to assume that fibrin gelation in samples M-MF16, M-MF32 and M-MF48 took place, from the first few seconds, in the presence of MagP-OH particle structures distributed throughout the biomaterial and oriented in the direction of the applied field. Our hypothesis for the formation of the thick fibrin stripes we observed is that these chain-like particle structures acted as condensation fibers for the braid of biopolymer fibers, so that only some residual fibers gelled outside the stripes, giving rise to the microscopic pattern seen in samples M-MF16, M-MF32 and M-MF48 (Fig 1H). This hypothesis is also supported by the fact that no MagP-OH nanoparticles were observed in Fig 1H, from which we infer that all the particles were trapped in the fibrin stripes.

In this connection, we note that according to Tampieri et al. [23], in scaffolds made of magnetic particles and hydroxyapatite–collagen composites, the magnetic phase acts as a cross-linking agent for the collagen. Furthermore, Panseri et al. [21] showed that the fibril network in scaffolds made of magnetic particles and hydroxyapatite–collagen composites was influenced by the preparation method. When the particles were already dispersed in the solution before polymer gelation started (as in the engineered biomaterials described here), the magnetic phase was completely amalgamated and homogeneously distributed throughout the fibril network. On the other hand, when the magnetic scaffold was obtained by soaking a previously prepared nonmagnetic scaffold in a ferrofluid, the nanoparticles were simply adsorbed onto the surface of the collagen fibers. Thanikaivelan et al. [35] found that the collagen fibers were considerably stabilized when superparamagnetic Fe_2_O_3_ nanoparticles suspended in a liquid carrier were used upon application of a magnetic field of approximately 2,000 Oe (approximately 160 kA/m).

### Cell viability analysis

**Fig 2:**
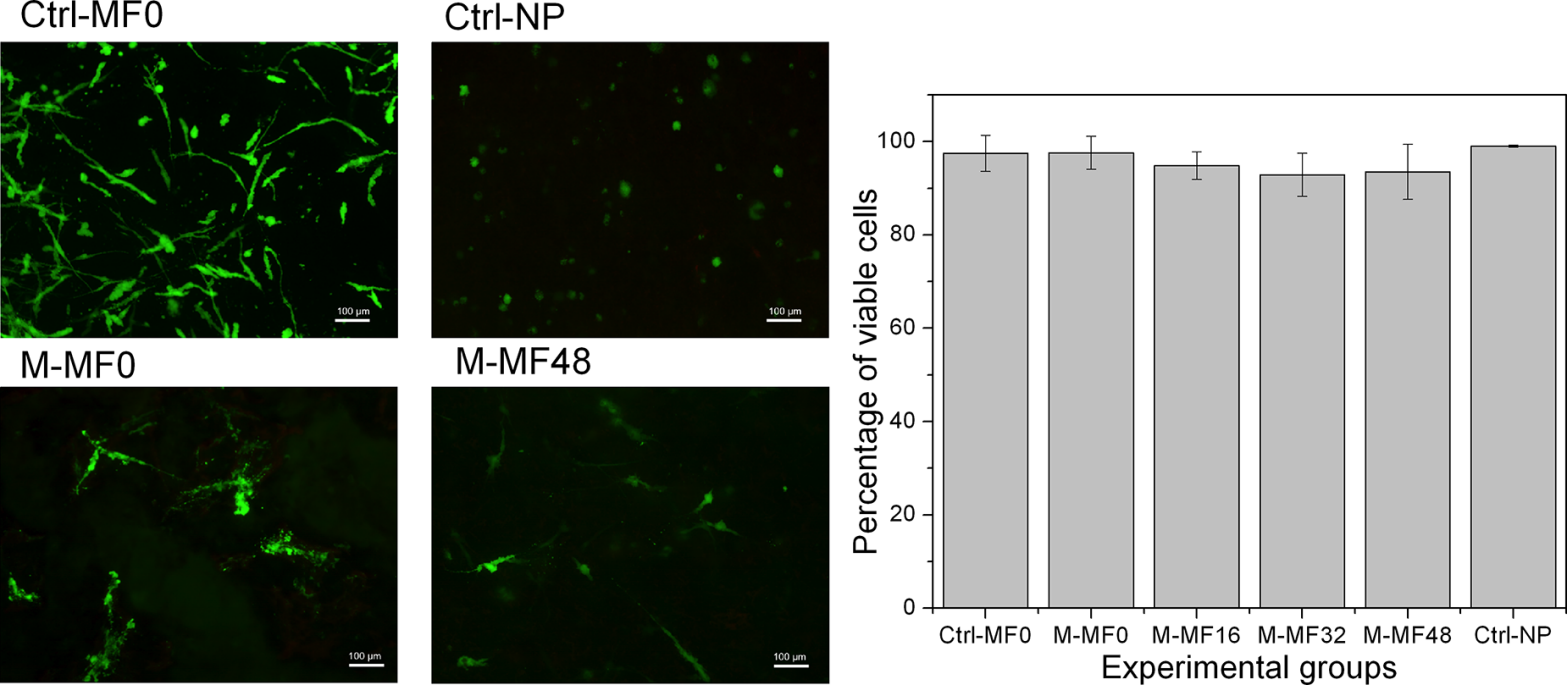
Cell viability tests. Fluorescence microscopy images (scale bar: 100 μm) representative of each experimental group. Live cells are stained green, and dead cells red. The graph shows the mean values ± standard deviations for live cells from 8 independent experiments for each experimental group. Ctrl-MF0: control (nonmagnetic) tissue substitute without particles, gelled in the absence of a magnetic field; Ctrl-NP: control (nonmagnetic) tissue substitute with nonmagnetic polymer particles; M-MF0: magnetic tissue substitute gelled in the absence of a magnetic field; M-MF16, M-MF32 and M-MF48: magnetic tissue substitutes gelled during application of a 16 kA m^-1^, 32 kA m^-1^ or 48 kA m^-1^ field, respectively.

Representative fluorescence microscopy images from the viability assays are shown in Fig 2. From these images we obtained the number of viable cells, which remained above 90% in all experimental groups, with no significant differences (p>0.05) among groups (Fig 2).

**Fig 3:**
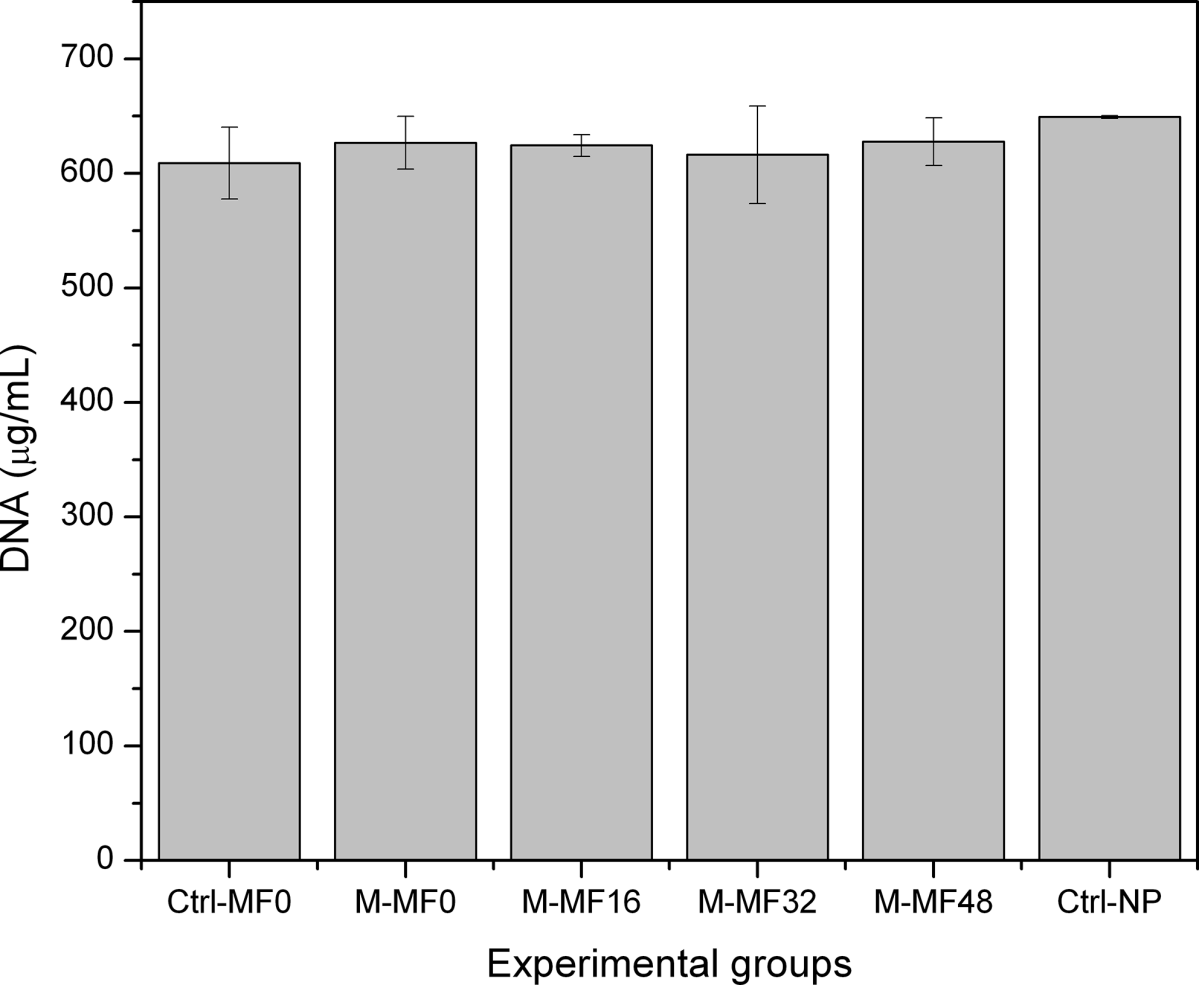
Quantification of DNA release. Integrity of the nuclear membrane was studied by quantifying the DNA released in the culture medium. The graph shows the mean values ± standard deviations of 8 independent experiments for each experimental group. Ctrl-MF0: control (nonmagnetic) tissue substitute without particles, gelled in the absence of a magnetic field; Ctrl-NP: control (nonmagnetic) tissue substitute with nonmagnetic polymer particles; M-MF0: magnetic tissue substitute gelled in the absence of a magnetic field; M-MF16, M-MF32 and M-MF48: magnetic tissue substitutes gelled during application of a 16 kA m^-1^, 32 kA m^-1^ or 48 kA m^-1^ field, respectively.

Similarly, we observed no significant difference (p>0.05) in the amount of free DNA among the different experimental groups (Fig 3).

According to results in Figs 2 and 3, cell viability was high in both magnetic and the nonmagnetic control tissue substitutes. We interpret this to mean that magnetic and nonmagnetic nanoparticles did not alter cell viability, and that magnetic tissue substitutes are likely to be safe for use in vivo, in agreement with previous results [12,13].

### Magnetic properties

**Fig 4:**
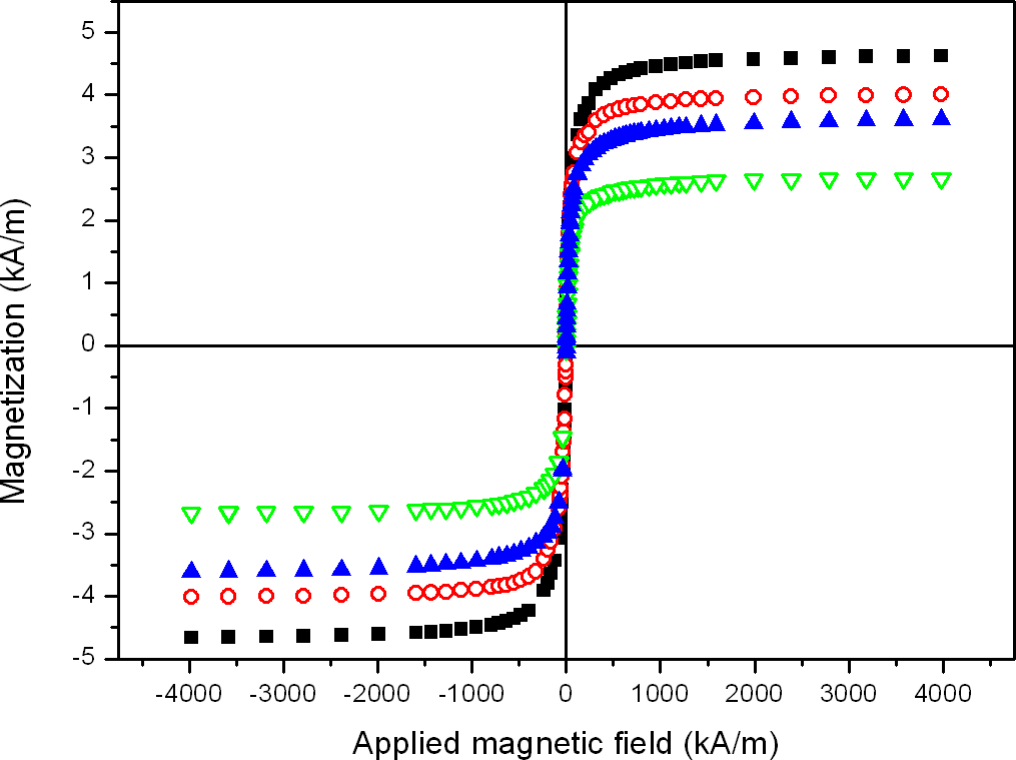
Magnetization curves of magnetic tissue substitutes. Filled squares: tissue substitute gelled in the absence of a magnetic field (M-MF0); open circles: tissue substitute gelled during application of a 16 kA m^-1^ field (M-MF16); open triangles: tissue substitute gelled during application of a 32 kA m^-1^ field (M-MF32); filled triangles: tissue substitute gelled during application of a 48 kA m^-1^ field (M-MF48). Values for saturation magnetization (kA/m) were obtained according to the Fröhlich–Kennely law [36]: M-MF0: 4.7 ± 0.3; M-MF16: 4.04 ± 0.24; M-MF32: 2.67 ± 0.15; M-MF48: 3.57 ± 0.20.

The magnetization curve of MagP-OH particles (not shown) displayed typical soft ferromagnetic features, with a saturation magnetization of 161 ± 7 kA/m, obtained by fitting the experimental data to the Fröhlich–Kennely law [36]. Similarly, magnetic tissue substitutes showed soft ferromagnetic features, although with much lower saturation magnetization values (Fig 4). Differences in the saturation magnetization values between different magnetic tissue substitutes were most likely due mainly to their different MagP-OH particle content. In fact, the concentration of MagP-OH particles in the tissue substitutes can be estimated by comparing their saturation magnetization (obtained by fitting the experimental data to the Fröhlich–Kennely law) to the saturation magnetization of MagP-OH powder, on the basis of the mixing law [37]. From the best fits to the mixing law, we obtained the following values for MagP-OH particle volume concentration (*ϕ*), M-MF0: 2.9 ± 0.3 vol%; M-MF16: 2.5 ± 0.3 vol%; M-MF32: 1.66 ± 0.16 vol%; M-MF48: 2.22 ± 0.22 vol%. Note that as expected, nonmagnetic control tissue substitutes did not show any ferromagnetic behavior.

### Mechanical properties

**Fig 5:**
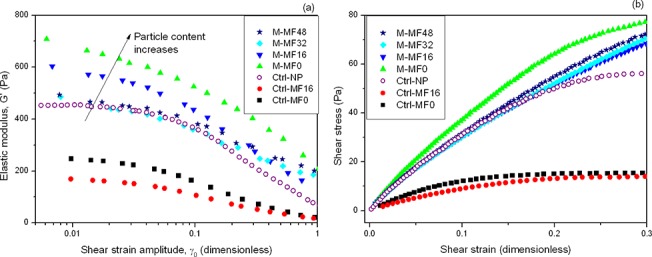
Mechanical properties of tissue substitutes in the absence of a magnetic field. (a) Elastic modulus as a function of the amplitude of shear strain in oscillatory tests at an oscillation frequency of 1 Hz. (b) Shear stress as a function of shear strain obtained under steady-state conditions. Experimental groups: Ctrl-MF0 and Ctrl-MF16: control (nonmagnetic) tissue substitute without particles, gelled in the absence of a magnetic field or during application of a 16 kA m^-1^ field, respectively; Ctrl-NP: control (nonmagnetic) tissue substitute with nonmagnetic polymer particles; M-MF0: magnetic tissue substitute gelled in the absence of a magnetic field; M-MF16, M-MF32 and M-MF48: magnetic tissue substitutes gelled during application of a 16 kA m^-1^, 32 kA m^-1^ or 48 kA m^-1^ field, respectively.

In the absence of a magnetic field, we observed much higher values for elastic modulus G′ (up to 4 times as high) and shear stress (up to 5 times as high for a given value of shear strain) in the oral mucosa tissue substitutes that contained either magnetic or nonmagnetic particles compared to tissue substitutes without particles (Fig 5).

The differences between the values for nonmagnetic control tissue substitutes without particles were small. On the other hand, tissue substitutes containing particles differed in G′ by as much as 40%, whereas shear stress was approximately similar in all samples with the exception of magnetic samples gelled in the absence of an applied field (M-MF0), and nonmagnetic control samples containing polymer particles (Ctrl-NP) at the highest strain values. Regarding the correlation between G′ and shear strain amplitude, we found that in all cases G′ showed an initial pseudoplateau at low amplitude, followed by a decrease at medium and high amplitudes. The initial pseudoplateau determines the so-called viscoelastic linear region (VLR) and the rest of the curve is referred to as the nonlinear viscoelastic region. Usually, the value of G′ pertaining to the VLR is considered an indicator of the strength of the material: the higher the G′, the stronger the material. With respect to the shape of the curves of shear stress vs. shear strain, we observed an initial linear portion at low strain values, where stress was proportional to strain. The proportionality constant is known as the shear modulus, G. At higher values of shear strain linearity was lost, and stress increased more slowly. Apart from the higher values of G for samples containing either magnetic or nonmagnetic particles compared to nonmagnetic control samples without particles (up to 3 times as high), we note that linearity was maintained up to much higher strain values in the former samples, especially magnetic tissue substitutes gelled during field application, compared to the nonmagnetic samples (Fig 5B). Note that G is also usually considered a measure of the strength of a material.

With respect to the differences in G′ among different magnetic samples, to analyze the effect of the concentration of magnetic particles on this value, we calculated the increase in G′ with respect to the average value in control tissue substitutes without particles (G′_control_) and normalized these values to the volume fraction of magnetic particles, *ϕ*_*MagP*−*OH*_, and G′_control_:

$$\frac{G'-G{'}_{control}}{\phi_{MagP-OH}\cdot G{'}_{control}}.$$ (1)

**Fig 6:**
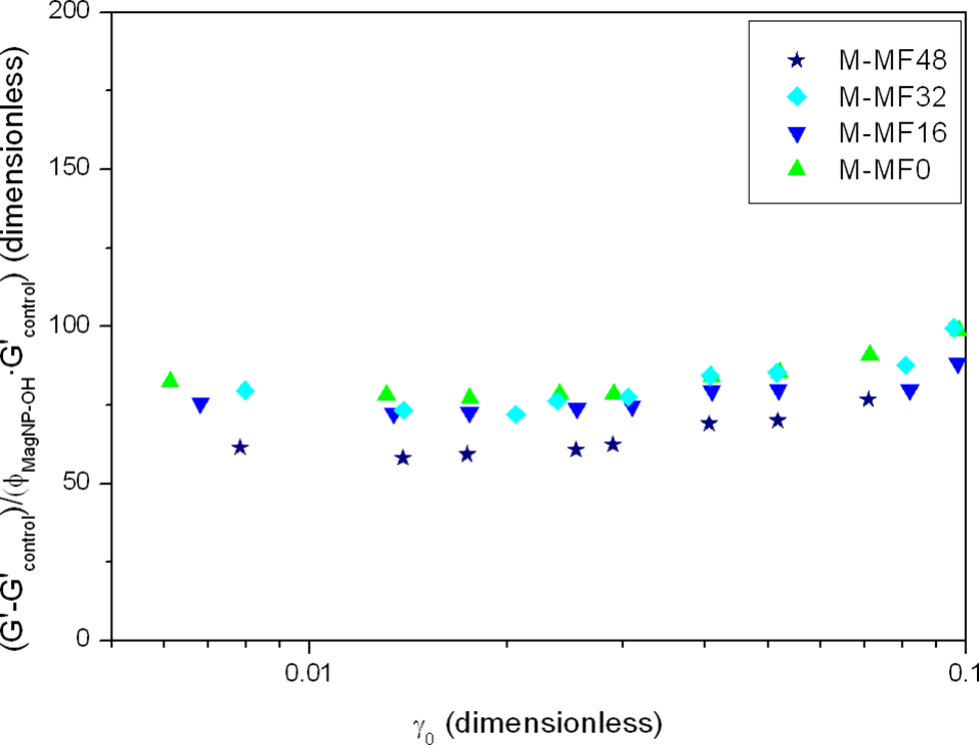
Normalized elastic modulus of magnetic tissue substitutes plotted as a function of shear strain amplitude. The elastic modulus, G′, of different magnetic tissue substitutes was normalized to account for the increase with respect to the elastic modulus of control (nonmagnetic) tissue substitutes without particles (Ctrl-MF0 to Ctrl-MF48), G′_control_, per unit volume concentration of magnetic particles, *ϕ*_*MagP*−*OH*_. Experimental groups: M-MF0: magnetic tissue substitute gelled in the absence of a magnetic field; M-MF16, M-MF32 and M-MF48: magnetic tissue substitutes gelled during application of a 16 kA m^-1^, 32 kA m^-1^ or 48 kA m^-1^ field, respectively.

The normalized G′ values approximately overlapped in a single master curve, as shown in Fig 6.

From the initial linear portion of the curves in Fig 5B we obtained the shear modulus (G) of the samples as the slope of the curves. As per our procedure for elastic modulus, we analyzed the effect of magnetic nanoparticles by defining a normalized shear modulus:

$$\frac{G-G_{control}}{\phi_{MagP-OH}\cdot G_{control}}.$$ (2)

Normalized shear modulus data are shown in Table 2. Although normalized shear modulus values differed among samples, they overlapped when experimental error was taken into account.

**Table 2:** Normalized shear modulus (Eq 2) of different magnetic tissue substitutes. G_control_ = 101 ± 10 Pa represents the mean value for control samples without particles (Ctrl-MF0 to Ctrl-MF48). Uncertainties were estimated according to theory of error propagation.

Sample name^a^	M-MF0	M-MF16	M-MF32	M-MF48
Normalized shear modulus: (dimensionless)	81 ± 21	49 ± 16	74 ± 22	58 ± 17

^a^ Experimental groups: M-MF0: magnetic tissue substitute gelled in the absence of a magnetic field; M-MF16, M-MF32 and M-MF48: magnetic tissue substitutes gelled during application of a 16 kA m^-1^, 32 kA m^-1^ or 48 kA m^-1^ field, respectively.

The results in Fig 6 and Table 2 indicate that the higher G′ and G in magnetic tissue substitutes than control samples without particles (Ctrl-MF0 to Ctrl-MF48) were approximately proportional to the volume concentration of magnetic particles. This result is consistent with the predictions of the classical theory of mechanics of composite materials for a continuous matrix with spherical inclusions [38]. Note that the higher G′ and G in control samples with nonmagnetic polymer particles (Ctrl-NP) compared to control samples without particles (Ctrl-MF0 to Ctrl-MF48) are assumed to be governed by the same theory. In particular, in the special case where the spherical inclusions are completely rigid and under dilute conditions, and the matrix material is incompressible, the classical theory of mechanics of composite materials predicts [38]:

$$G=\left(1+2.5\phi \right)G_{c}\Rightarrow \frac{G-G_{c}}{\phi G_{c}}=2.5$$ (3)

where G_c_ is the shear modulus of the continuous matrix. The quotient in Eq (3) has the same structure as the normalized shear modulus defined by Eq (2), where G_control_ is replaced by G_c_. The value of 2.5 in Eq (3) can thus be interpreted as the theoretical value predicted by the classical theory of mechanics of composite materials for the normalized shear modulus. It is therefore informative to compare this theoretical value of 2.5 against the experimental normalized shear modulus (Table 2). As observed, the normalized shear modulus of magnetic tissue substitutes was much higher than 2.5, which can be taken as evidence of the much stronger structure of the continuous matrix of magnetic tissue substitutes compared to control tissue substitutes without particles. In fact, Eq (3) can be used to calculate the shear modulus of the continuous matrix of magnetic tissue substitutes (Table 3).

**Table 3:** Shear modulus of the continuous matrix of magnetic tissue substitutes, as calculated with Eq (3). Note the mean value of the shear modulus in control samples without particles (Ctrl-MF0 to Ctrl-MF48), G_control_ = 101 ± 10 Pa. Uncertainties were estimated according to the theory of error propagation.

Sample name^a^	M-MF0	M-MF16	M-MF32	M-MF48
Shear modulus of continuous matrix (Pa)	315 ± 4	211 ± 3	217 ± 3	218 ± 3

^a^ Experimental groups: M-MF0: magnetic tissue substitute gelled in the absence of a magnetic field; M-MF16, M-MF32 and M-MF48: magnetic tissue substitutes gelled during application of a 16 kA m^-1^, 32 kA m^-1^ or 48 kA m^-1^ field, respectively.

As shown in Table 3, there was a twofold increase in the shear modulus of the continuous matrix in magnetic tissue substitutes gelled during exposure to a magnetic field, compared to control tissue substitutes without particles. In magnetic tissue substitutes gelled without a magnetic field, the shear modulus of the continuous matrix was even higher, with a threefold increase compared to control tissue substitutes. These enhancements in the mechanical properties of the continuous matrix when magnetic particles were included in the formulation of the engineered tissue substitutes may be due to the changes in the microscopic pattern of the fibrin network induced by the magnetic particles. The same argument would apply for the enhanced mechanical properties of control tissue substitutes containing nonmagnetic polymer particles (Ctrl-NP) compared to control tissue substitutes without particles (Ctrl-MF0 to Ctrl-MF48). These microstructural changes were evident in samples that were gelled during exposure to a magnetic field (M-MF16, M-M32, M-MF48), with thick stripes containing closely packed fibrin fibers aligned in the same direction, as discussed above. Changes in the microscopic pattern of the continuous matrix were not so intense in magnetic tissue substitutes gelled without application of a magnetic field (M-MF0) or in control tissue substitutes containing nonmagnetic polymer particles (Ctrl-NP); in both cases the likely reason for the enhanced mechanical properties is bonding and amalgamation of the fibers to the homogeneously distributed nanoparticles.

**Fig 7:**
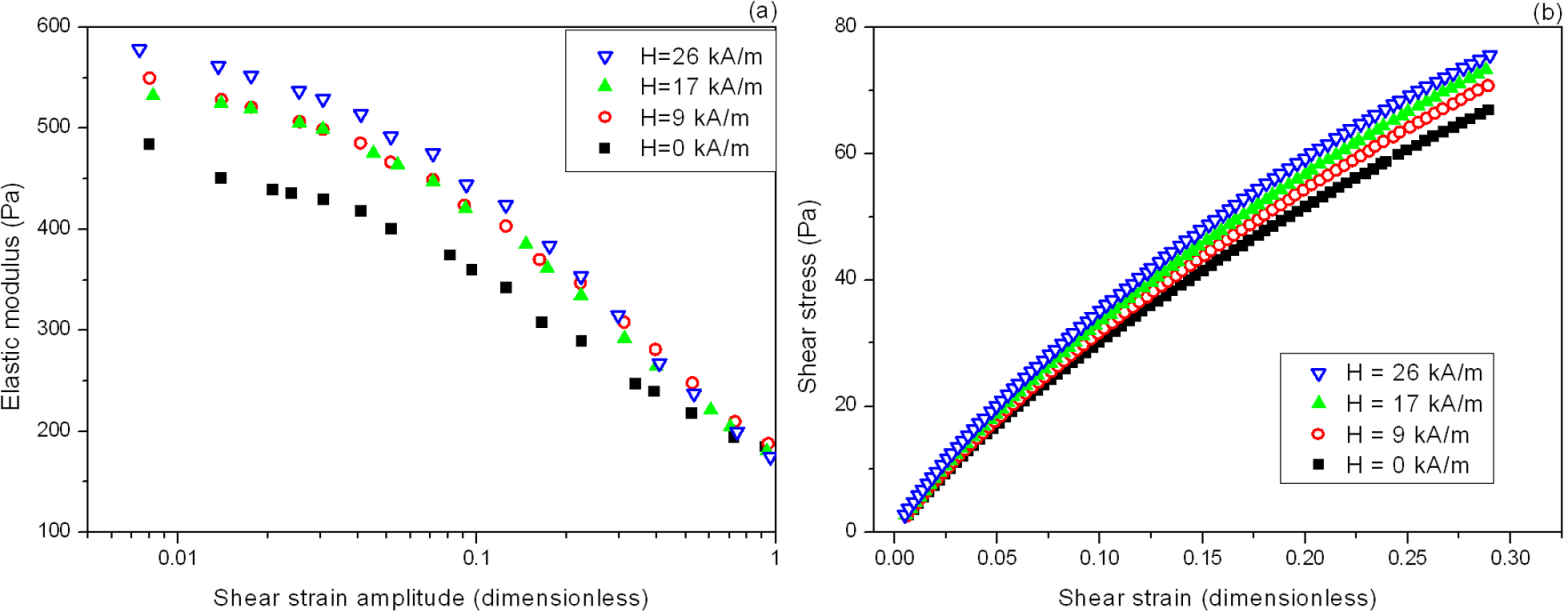
Effect of a magnetic field on the mechanical properties of magnetic tissue substitutes. Sample M-MF32 is a magnetic tissue substitute gelled during application of a 32 kA m^-1^ field. The effect of a magnetic field applied during measurement on the mechanical properties of the tissue substitute is shown as (a) the elastic modulus as a function of shear strain amplitude, and (b) the initial portion of the shear stress vs. shear strain curves. The intensities (*H*) of the magnetic field applied are shown.

With regard to the influence of magnetic field intensity during the rheological measurements, we observed–as expected–no effect in nonmagnetic control tissue substitutes. In M-MF0 tissue substitutes, there was little difference in the values of G′ (not shown). For these samples the effect on shear stress (results not shown) was larger, with a clear tendency of shear stress to increase with strength of the field applied. For M-MF16, M-MF32 and M-MF48 tissue substitutes, the magnetic field applied during rheological measurements had a notable effect on both G′ and shear stress values, as illustrated in Fig 7. We note that as described in the Material and Methods section, each point in this figure is the average of at least 9 measurements, including at least 3 successive cycles of increasing the magnetic field from 0 kA/m to 26 kA/m and then decreasing it again to 0 kA/m. Since we found no statistically significant differences among values for the same sample and field strength, we infer that the changes in mechanical properties after application of a magnetic field are reversible.

As observed, the shape of the G′-vs.-amplitude curves remained similar as the intensity of the magnetic field increased, despite the fact that G′ increased in average terms together with the strength of the magnetic field. The same was true for the shear stress-vs.-shear strain curves. From the linear portion of these curves we obtained the values of shear modulus, and observed a clear tendency for G to increase with the strength of the magnetic field applied in all magnetic tissue substitutes (Table 4).

**Table 4:** Effect of the magnetic field applied during measurement on the shear modulus (Pa) of magnetic tissue substitutes. Data in this table correspond to the best linear fit including experimental uncertainties.

Magnetic field strength (kA/m)	Sample M-F0 ^a^	Sample M-MF16 ^a^	Sample M-MF32 ^a^	Sample M-MF48 ^a^
0	338 ± 3	224 ± 3	225.8 ± 2.2	230 ± 3
9	363 ± 3	237 ± 3	243.6 ± 2.1	237 ± 3
17	369 ± 4	246 ± 3	239.6 ± 1.9	252 ± 3
26	370 ± 4	253 ± 3	245.0 ± 1.9	254 ± 3

^a^ Experimental groups: M-MF0: magnetic tissue substitute gelled in the absence of a magnetic field; M-MF16, M-MF32 and M-MF48: magnetic tissue substitutes gelled during application of a 16 kA m^-1^, 32 kA m^-1^ or 48 kA m^-1^ field, respectively.

We note that the increases in characteristic mechanical parameters (shear modulus, elastic modulus, Young modulus, etc.) in samples exposed to increasingly strong magnetic fields is typical of dispersions of multi-domain magnetic particles in a polymer matrix [30]. This phenomenon is known as the magnetorheological (MR) effect, and we refer to these systems as MR gels and MR elastomers. In fact, the magnitude of the increases we observed in shear modulus and elastic modulus with increasingly intense magnetic fields in magnetic tissue substitutes agrees well with previous research on MR elastomers. For example, Jolly et al. [39] found a maximum increase in shear modulus of 30% upon application of a magnetic field in an MR elastomer consisting of 10 vol% iron particles dispersed in a silicone-based polymer matrix. More recently, Ge et al. [40] reported a 43% increase in an MR elastomer consisting of approximately 7 vol% iron particles dispersed in natural rubber. The enhancements reported here were weaker most probably because of the lower concentration of magnetic particles in the polymer matrix and the weaker magnetic properties of magnetite (the main constituent of MagP-OH nanoparticles) compared to iron. Note, for example, that shear modulus increased by 10% in our magnetic tissue substitutes exposed to a 26 kA/m field compared to no exposure to a magnetic field during measurement, and elastic modulus showed a slightly large increase.

Finally, it is worth noting that both the magnetic and nonmagnetic tissue substitutes reported here had values of G and G′ on the same order of magnitude as those obtained in previous studies of fibrin–agarose scaffolds and oral mucosa tissue substitutes [31,33,41]. Moreover, G and G′ values were within the range of values reported for native human soft tissues (G ≈ 5–2500 Pa, G′ ≈ 10–5000 Pa) [31].

## Conclusions

We report a straightforward, versatile method for the preparation of a new type of tissue-engineered biomaterial characterized by the inclusion of multi-domain magnetic particles in a biopolymer matrix. Cell viability analyses of oral mucosa fibroblasts showed no significant differences in comparison to control (nonmagnetic) tissue substitutes of proven applicability in tissue regeneration. Thanks to their magnetic behavior, these novel tissue substitutes could be visualized and followed for in vivo applications by magnetic resonance imaging, and also act as magnets, attracting functionalized magnetic nanoparticles injected close to them. Although these advantages are also shared by other magnetic scaffolds described previously, a unique feature of our magnetic tissue substitutes is that their mechanical properties can be tuned in a controlled, reversible manner by noncontact magnetic force fields. Furthermore, we found that in the off state (absence of an applied field) the strength of our engineered magnetic tissue substitutes is also affected by the concentration of particles and other technical details, such as the application of a magnetic field during gelation. This versatility could be exploited in clinical applications to match the mechanical properties of tissue substitutes to those of natural target tissues. Several other potential advantages can be envisaged for our magnetic tissue substitutes, such as their adhesion by magnetic attraction in tissue replacements, which would reduce the need for surgical sutures in (for example) treatments for gingival recession. To conclude, we foresee that other similar field-responsive biological tissue substitutes will be generated in the near future, as applied research contributes to the development of a broad range of promising novel applications for smart magnetic biomaterials.

## Supporting Information

S1 Video Magnetic tissue substitute attracted by a magnetic field. The novel magnetic field-responsive tissue substitutes can be moved and manipulated by a noncontact magnetic force induced by a magnet.
(AVI)
